# Processing of prosodic cues of uncertainty in autistic and non-autistic adults: a study based on articulatory speech synthesis

**DOI:** 10.3389/fpsyt.2024.1347913

**Published:** 2024-10-14

**Authors:** Charlotte Bellinghausen, Bernhard Schröder, Reinhold Rauh, Andreas Riedel, Paula Dahmen, Peter Birkholz, Ludger Tebartz van Elst, Thomas Fangmeier

**Affiliations:** ^1^ Institute of German Studies, University of Duisburg-Essen, Duisburg, Germany; ^2^ Department of Child and Adolescent Psychiatry, Psychotherapy, and Psychosomatics, Medical Center – University of Freiburg, Faculty of Medicine, University of Freiburg, Freiburg, Germany; ^3^ Department of Psychiatry and Psychotherapy, Medical Center – University of Freiburg, Faculty of Medicine, University of Freiburg, Freiburg, Germany; ^4^ Luzerner Psychiatrie, Ambulante Dienste, Luzern, Switzerland; ^5^ Institute of Acoustics and Speech Communication, Technische Universität Dresden, Dresden, Germany

**Keywords:** speech perception, autism spectrum disorder, prosody, uncertainty, emotion perception, theory of mind

## Abstract

**Introduction:**

We investigated the prosodic perception of uncertainty cues in adults with Autism Spectrum Disorder (ASD) compared to neurotypical adults (NTC).

**Method:**

We used articulatory synthetic speech to express uncertainty in a human-machine scenario by varying the three acoustic cues *pause*, *intonation*, and *hesitation*. Twenty-eight adults with ASD and 28 NTC adults rated each answer for uncertainty, naturalness, and comprehensibility.

**Results:**

Both groups reliably perceived different levels of uncertainty. Stimuli were rated as less uncertain by the ASD group, but not significantly. Only when we pooled the recipients’ ratings for all three cues, did we find a significant group difference. In terms of reaction time, we observed longer reaction times in the ASD group compared to the neurotypical comparison group for the uncertainty level hesitation & strong intonation, but the differences were not significant after Bonferroni correction. Furthermore, our results showed a significant group difference between the correlation of uncertainty and naturalness, i.e. the correlation in the ASD group is significantly lower than in the NTC group. Obtained effect size estimates can inform sample size calculations in future studies for the reliable identification of group differences.

**Discussion:**

In future work, we would like to further investigate the interaction of all three cues and uncertainty perception. It would be interesting to further vary the duration of the pause and also to use different types of fillers. From a developmental perspective, uncertainty perception should also be investigated in children and adolescents with ASD.

## 1 Introduction

We present an empirical study investigating the perception of uncertainty cues in adults with ASD compared to the NTC group. To generate our material, we used articulatory speech synthesis with varying prosodic uncertainty features. The utterances were presented to the participants and they were asked to rate them. We consider the ascription of (un)certainty as a part of affective ToM and assume that (u)certainty can be expressed prosodically without interaction with syntactic or semantic features of an utterance. Thus, its effect can be studied in isolation. In the following introductory section, we provide the theoretical background of our research goal and outline the state of research on the role of prosody perception in ASD. This includes studies of emotion perception, speech synthesis perception, and uncertainty perception in both human-human and human-machine interaction.

According to DSM-5 (1) and ICD-11 (2) Autism Spectrum Disorder (ASD) is classified as a neurodevelopmental disorder with severe impairments in the domains of social communication and restrictive repetitive behaviors/interests. The prevalence is approximately 1% (3, 4). The male-female ratio in well-ascertained epidemiological samples is about 3:1. However, there are concerns about under-reporting in girls and women [cf. (1): 64]. The etiology of ASD shows a strong genetic component as well as other causes (4).

In this article we will focus on Autism Spectrum Disorder without accompanying intellectual impairment (ASD without II). A description of ASD without II can be found, for example, in Riedel (5) and in Vogeley (6). In the area of language processing, syntactic and semantic processing are barely affected in ASD without intellectual impairment taking into account the semiotic dimensions according to Morris (7), but problems in pragmatic interpretation are often found [ (8): 4f.]. For example, adults with ASD without II often have difficulty understanding non-lexicalized metaphors as assessed by the Freiburg Questionnaire of Linguistic Pragmatics (FQLP) (9). Although in the literature, often problems in general pragmatic processing in ASD without intellectual impairment are described, it has been shown that the pragmatic abilities for hearers with ASD without II differ between pragmatic domains [cf. (10): 114, see also (11)]. In terms of syntactic processing, Durrleman et al. (12) tested relative clause comprehension in autistic participants with and without reported language delay. They found that the participants with reported language delay had more difficulty with subject relatives than those without language delay. It should be noted here that we assume that syntax is more likely to be impaired in autistic individuals with delayed language development. However, in the case of autism without intellectual impairment pragmatics is the focus of our research interest.

Several empirical studies investigated the prosodic competence of participants with ASD without II. The term prosody is defined as “[ … ] a set of higher-level organizational structures that account for variations in pitch, loudness, duration, spectral tilt, segment reduction and their associated articulatory parameters” [ (13): 327].

At the interface of syntax and pragmatics, the work of Martzoukou et al. (14, 15) suggested evidence of problems with the use of prosody in syntactic processing. Similarly, Terzi et al. (16) reported difficulties at the interface of morpho-syntax with pragmatics and prosody in ASD without intellectual impairment.

However, several studies have focused on the perception and production of prosody and its signaling of pragmatic and emotional features of utterances. In the following, we present selected previous studies that have investigated the role of prosody in both speech production and speech perception in order to place our empirical study in a theoretical context. Since prosody has different linguistic and paralinguistic functions [cf. (17): 326], we refer first to linguistic functions such as the marking/perception of information status, i.e. structural prosodic functions [for an overview see (18)]. For example, prosody can be used to indicate the information status of a sentence (19). Afterwards we will discuss paralinguistic functions of prosody such as emotion expression/perception, i.e. affective prosodic functions [for an overview see (20)]. An overview of linguistic prosody in ASD is given in Grice et al. (21). In this work, the various functions of prosody are described in more detail. Depending on the prosodic function, there are differences between the ASD and NTC (neurotypical control) groups.^1^

In terms of structural prosody skills in ASD, Shriberg et al. (22) reported for accentuation that speakers with ASD without II aged 10-50 years were less likely to use stress and phrasing appropriately compared to NTC. Similarly, Paul et al. (23) reported difficulties in stress production and also in speech perception more often in the ASD group without II compared to the NTC in speakers aged 14-21 years. In addition, Kiss et al. (24) found significant differences in global pitch distribution comparing children aged 4 to 9 years and a NTC group using the CSLU Autism Speech Corpus. Nadig and Shaw (25) observed a higher pitch range in speakers aged 8-14 years old with ASD without II in contrast to the NTC, but neurotypical students did not rate this as increased pitch variation. Wehrle et al. (26) also found a tendency for adults with ASD to have a higher pitch range compared to the NTC. For both prosody perception and production, Diehl and Paul (27) showed that children and adolescents aged 8-16 years with ASD without II required more time to imitate intonation patterns than the NTC.

For adults with ASD without II, the perception study by Grice et al. (28) suggested evidence that adults with ASD showed a reduced sensitivity to intonation and consequently based their judgments less on the word pronunciation in comparison to neurotypical adult hearers. Instead, word frequency was more important than intonation for decoding of information structure (i.e. the division of sentences into new and known information) in autistic hearers. In contrast, Globerson et al. (29) found no differences between adult hearers with and without ASD using prosody for pragmatic focus interpretation (i.e. the detection of new information in a sentence)^2^. The groups also did not differ in psychoacoustic tests. In contrast to that, the group with ASD performed less accurately on both the acoustic prosody recognition task and the facial emotion recognition task.

In their systematic review of linguistic prosody in ASD, Grice et al. (21) examined both production and perception of prosodic functions in grammar and pragmatics, as well as emotion. They categorize prosodic functions on a scale of more “formal” (rule-based) functions and more “intuitive” (highly context-dependent functions). Lexical stress, lexical tones and grammatical functions of prosody belong to the most formal functions, marking of intentions and emotions to the most intuitive [cf. (21): 2-5]. The results for perception suggested that the more intuitive aspects of prosody are more difficult in ASD, i.e. perceiving information status, intention, and emotional state. In contrast, the more formal aspects of prosody such as lexical and syntactic functions appear to be relatively unaffected [cf. (21): 6-8]. No clear overarching pattern was found for prosody production [cf. (21): 12]. However, there was a tendency for differences in general prosodic characteristics in speech production [see (21):13].

In conclusion, the results of the presented studies on structural prosody in ASD are not clear with respect to group differences. This can be explained by the different functions of prosody. Prosodic uncertainty marking is not one of the ‘formal aspects’ of prosody and we would therefore expect to see stronger differences between groups.

After reviewing previous studies of prosody production and perception in hearers with ASD without II, we turn to affective prosody skills in ASD and refer to previous work on the expression and recognition of emotion in ASD. The reason for this is that emotions and epistemic states can also be expressed through prosody [cf. (32): 48]. This is relevant to our current experimental study in which we express different degrees of intended uncertainty by means of prosody using articulatory speech synthesis (33)^3^.

In their Facial Recognition Task, Doi et al. (34) generated varying degrees of anger, happiness, and sadness, as well as a neutral face. In the Emotional Prosody Recognition Task, a naturally spoken Japanese utterance was presented in an angry, happy, and sad way of speaking at different intensities Also a neutral acoustic stimulus was used [cf. (34): 2102 ff.]. The adults in the ASD group performed worse at recognizing angry and sad faces and voices. There was an effect of emotional intensity on emotion recognition. For facial expression recognition, there was a lower recognition in the ASD group compared to the NTC group for the stimuli of intermediate emotional intensities [cf. (34): 2109].

Hsu and Xu (35) used the articulatory speech synthesizer VocalTractLab (36) to produce modal, breathy, and pressed voices in Mandarin. Hearers with ASD without II and a NTC were asked to judge body size, emotion (happiness, anger, and neutral emotion) and attitude [cf. (35): 1925]. The results showed that the adolescents with ASD were less sensitive to auditory manipulation than their neurotypical peers [cf. (35): 1927]. However, to our knowledge, uncertainty perception has not been investigated using articulatory speech synthesis. We will use this type of speech synthesis to model uncertainty and test its influence on uncertainty perception in our empirical study.

In two meta-analyses of facial emotion recognition (37, 38), participants with ASD showed significantly poorer performance in recognizing basic emotions compared to the NTC group for a subset of basic emotions. However, Scheerer et al. (39) found that autistic and typically developing children were accurate in matching emotional voice clips to emotion words, but autistic children had difficulty in matching emotional voice clips to emotional faces. Lartseva et al. (40) likewise document the presence of impairments in emotional language processing in individuals with ASD. These appear to be fairly independent of stimulus complexity, task complexity, and sensory modality as well as the level of language development. Lui et al. (41) investigated the role of psychoacoustic abilities in affective prosody recognition in autistic adults. Their results indicated that psychoacoustic abilities were used as a compensatory mechanism for deficits in higher-order processing of emotional signals in social interactions.

In our recent study (42) we presented a systematic analysis of 12 selected studies on emotion perception for the auditory and/or visual modality. The analysis revealed that in most cases basic emotions according to Ekman (43) were tested exclusively or in combination with complex emotions. The results generally showed a difference in perception between the ASD and NTC groups for the different modalities with only two studies showing no difference in visual emotion perception.

In their systematic review of affective prosody recognition in ASD concerning basic emotions according to Ekman (43), Zhang et al. (44) investigated potential factors for differences in study results comparing ASD and NTC groups. Their results showed that the level of difficulty in affective prosody recognition experienced by hearers with ASD varied across basic emotions.

As the aforementioned studies on emotion perception in ASD have shown divergent results regarding differences in emotion processing between autistic and non-autistic hearers, we believe that further research is needed in this area. The studies mentioned above have in common that mainly basic emotions according to Ekman (43) were investigated. In our work, we focus on uncertainty as a non-prototypical emotion. To our knowledge, there is a research gap regarding the perception of uncertainty in ASD. With our study, we hope to contribute to the understanding of how uncertainty is processed as a non-prototypical emotion by hearers with ASD and thus fill this research gap.

Next, we will further motivate why we consider the perception of uncertainty conveyed by prosodic cues in ASD to be a particular interest. We assume that uncertainty refers to the statement in the utterance of the prosodic information. The speaker’s belief state, including the perceived uncertainty, is part of the hearer’s ToM [see Theory of Mind; (45)]. We regard the attribution of uncertainty to another person, i.e. the speaker, as a case of *affective* ToM, but with reference to a *proposition* (a statement or a fact about the speaker is uncertain), i.e. to a conceptual content.

Uncertainty could therefore be understood as an affective propositional attitude. In philosophy, psychology, linguistics, and cognitive science, propositional attitudes are understood as the mental phenomena expressed by sentences such as *Galileo believes that the earth moves* and *Pia hopes that it will rain* (i.e. the belief about the movement of earth and the hope of rain). Even if propositional attitudes are discussed critically, it is agreed that they are mental phenomena and play a central role in our everyday practice of describing, explaining, and predicting others and ourselves [cf. (46)]. Even basic emotions according to Ekman (43) such as fear or surprise can be attitudes towards propositions, e.g. *fearing that one shall be killed in an avalanche* or *being surprised that New York is further south than Rome.* A discussion of propositional attitude approach to emotions is given in Cudney (47). According to Giannakidou and Mari (48), emotion attitudes appear as gradable psychological attitudes, i.e. *be happy*, *be surprised*, *be angry*, and are assumed to be factive.

In the field of prosody research, Hirschberg [ (49): 532] notes that the variation in prosody influences the interpretation of linguistic phenomena in many languages. Speakers can also use prosody to indicate the propositional attitude they have towards a certain proposition when uttering a sentence expressing that proposition [see also (49): 532].

As already noted above, uncertainty is a complex phenomenon. When we refer to uncertainty we mean uncertainty in answers in question-answer situations as will be explained below. Thus, the aim of our study is to empirically investigate the perception of uncertainty in autistic hearers in order to get a broader picture of emotion processing in ASD.

Next, we will explain the theoretical background of the communication of uncertainty in face-to-face communication in neurotypical hearers. Then we will further explain the motivation for our empirical investigation in hearers with ASD.

### 1.1 Communication of uncertainty

The expression and perception of uncertainty is essential in communication [cf. (50): 8]. As remarked in Wollermann [ (51): 80f.], uncertainty can generally be regarded as a non-prototypical emotion [see also (52)]. Kuhltau (53) categorizes uncertainty in cognitive terms. Furthermore, uncertainty can be considered from an epistemic point of view in communication (54). A discussion of whether epistemic emotions are metacognitive can be found in Carruthers (55).

Following Wollermann [ (51): 80], we assume that speakers and hearers communicate uncertainty in question-answer situations: communication partner A asks communication partner B a question. B is uncertain about the answer and expresses this uncertainty. A uses these uncertainty cues to decode B’s utterance and concludes that B is uncertain [cf. (51): 80]. It should be noted explicitly here that uncertainty is a complex phenomenon that encompasses different dimensions and definitions [see also (56): 138]. However, as noted above, we focus on uncertainty in responses to questions in communicative situations. We begin by referring to previous studies that have investigated the production and perception of uncertainty. We then discuss ideas of ToM and relate them to ASD in order to provide the theoretical background for our empirical study of uncertainty perception in ASD.

Smith and Clark (57) used the *Feeling of Knowing* paradigm following Hart (58) in order to test memory processes in adults in question-answering situations. Empirical results showed that uncertainty was marked, among other cues, lexically by the use of phrases such as “I guess” and by fillers such as “uh” and “um”. On the prosodic level pauses and rising intonation were observed as prosodic indicators of uncertainty [cf. (57): 32ff., see also (51): 82f.]. In order to test the perception of another speaker, Brennan and Williams (59) defined the *Feeling of Another’s Knowing* paradigm. They reproduced the study of Smith and Clark (57). In a further step, they used the audio material for listening evaluation. It was found that lexical hedges, rising intonation and delay contributed to the perception of uncertainty [cf. (59): 383; see also (51): 83]. Swerts and Krahmer (60) investigated the production and perception of uncertainty in the audio, visual, and audiovisual conditions. Uncertainty in answers was recognized in all three conditions, but recognition was easier in the audiovisual condition than in the unimodal conditions.

From a developmental perspective, Krahmer and Swerts (61) tested 7-8 year old neurotypical children and adults for the perception and production of uncertainty in question-answer situations in audiovisual speech. Uncertain utterances produced by adult speakers were recognized more accurately than children’s uncertain utterances by both children and adults as hearers. In addition, adults performed better than children in the recognition of uncertainty.

After referring to studies on uncertainty perception and production, we now provide the relevant background on ToM for our empirical study. Premack and Woodruff [ (45): 515] define ToM as follows: “An individual has a theory of mind if he imputes mental states to himself and others”. The concept of ToM also known as “mind reading” refers to the understanding of one’s own thoughts and feelings and those of others, and is central for human social interaction and communication. There is empirical evidence that it develops very early in human ontogeny [cf. (62): 1357]. An overview of ToM can be found, for example, in Astington and Dack (63) and in Leslie (64).

According to Kamp-Becker and Bölte [ (65): 40], children with ASD often have serious problems executing theory of mind tasks. In their seminal work, Baron-Cohen and et al. (66) discussed whether the autistic child has a ToM. Their study and that of Happé (67) suggested that children with ASD had problems in passing false-belief-tasks.^4^ However, it has to be discussed critically if a general ToM deficit occurs in individuals with ASD. As Chevallier [ (70): 4825] remarks there is evidence that there are problems related to ToM in ASD on the basis of standard false belief tasks or other more fine-grained tests. However, the characteristics of these impairments are still debated, i.e. if it is a primary or simply consecutive to more basic deficits [cf. (70): 4825]. Furthermore, the study by Tager-Flusberg (71) suggested that autistic participants who had passed a standard test with first-order false belief tasks, were even able to solve more complex second-order belief tasks when processing demands were reduced. In addition, the work of Iao and Leekam (72) showed that difficulties with the false representation tasks in children with ASD could not be explained by executive functions or language impairments. This may provide evidence to support the position that children with ASD may not have a specific theory of mind deficit.

As Gabriel et al. [ (69): 534] pointed out, ToM is a complex phenomenon that can be divided into cognitive and affective ToM [e.g., (73)]. On the one hand affective ToM refers to the representation of implications about emotions. On the other hand cognitive ToM is a term that describes implications about knowledge, intentions, and beliefs [cf. (69): 534]. For early adolescence, there was a correlation between both types of ToM and attention. There was also a correlation between cognitive ToM and language comprehension on the one hand, and a correlation between affective ToM and verbal intelligence, verbal fluency, and verbal flexibility. In middle and late adolescence, both types of ToM were correlated with affective intelligence. On the other hand, there was a correlation between cognitive ToM and working memory, figural intelligence, and language comprehension. Thus, the results for cognitive and affective ToM showed a developmental step in middle adolescence. There were also gender differences in cognitive ToM [cf. (69): 533].

Raimo et al. (74) investigated both types of ToM in neurotypical individuals during adulthood. According to Raimo et al. [ (74): 10], the decline of the affective component of ToM occurs earlier in adulthood (from the age of 60) than the cognitive component (from the age of 70). This decline in the first age group is related to the ability to infer others’ emotions and to decode emotional expressions in the nonverbal modality, rather than to the ability to infer emotional mental states from social stories in the verbal modality. In the older group, the decline is independent of the verbal or nonverbal modality of the task used [cf. (74): 10].

It should be noted that these two subtypes of ToM, i.e. affective vs. cognitive ToM, are not always clearly distinguished. The demarcation is not always consistent and is not always sharp. We talk about needs which have rather an emotional component, e.g. when there is a need for getting comfort, or a cognitive character, e.g. when we are curious about something.

We now turn to previous studies of affective and cognitive ToM in ASD. Begeer et al. (75) investigated affective ToM and tested children’s understanding of emotions based on counterfactual reasoning.^5^ The autistic children had problems in explaining emotions based on downward counterfactual reasoning (i.e. contentment and relief) compared to the neurotypical children. In contrast, there were no group differences in emotions based on upward counterfactual reasoning (i.e. disappointment and regret). The results also showed a relationship between second-order false-belief reasoning and children’s understanding of second-order counterfactual emotions for the neurotypical comparison group. However, children with ASD were more likely to rely on their general intellectual abilities [cf. (75): 301].

Scheeren et al. (77) tested comprehension of social stories containing second-order false belief display rules, double bluff, faux pas, and sarcasm. They found that children and adolescents with ASD performed as well as the NTC group. The age effect was consistent with adolescents performing better than children. Success on advanced ToM tasks was also determined by age, verbal abilities, and general reasoning abilities.

Similarly, Kimhi’s (78) review showed that language and verbal abilities, as well as general reasoning, facilitated better ToM comprehension in ASD [cf. 78: 340]. They also noted that ToM is a critical factor in children’s socio-cognitive development (cf. (78): 339).

There is currently some debate as to whether or not the feeling of uncertainty (and its supposed opposite, the feeling of certainty) belongs specifically to the category of so-called “epistemic emotions” in particular or can be considered as an emotion at all [see Meylan (79) for a con position, and Silva (80) for a pro position]. Whatever its exact nature, there is broad agreement that the feeling of uncertainty is an affective mental state. For example, Morriss et al. [ (81): 2] emphasize that “current theoretical models posit that uncertainty is aversive in and of itself and is consequently more likely to engage the behavioral inhibition system responsible for stress and associated negative emotional states, particularly anxiety and fear” [for a more detailed discussion see Morriss et al., (81)]. Consistent with this, the glossary of mental state terms in the well-known Reading-the-Mind-in-the-Eyes test (82, 83), which participants are asked to consult when they are unsure of the meaning of a response option, recurs on the concepts of feelings of certainty and uncertainty.

Andres-Roqueta and Katsos (11) investigated pragmatic skills in children with and without ASD. The tasks consisted of a linguistic-pragmatics task requiring competence with structural language and a social-pragmatics task requiring competence with ToM. They reported similar performance on structural pragmatics between the group with ASD and the NTC, but a lower performance on social pragmatics, which the authors explain with difficulties in ToM [cf. (11): 1494].

At this point, we would also like to address the link between ToM and compensation strategies [e.g. (84, 85)]. Livingston et al. [(84): 102] give the following example for compensation strategies: If a difficulty in distinguishing lies from jokes is masked by copying the behavior of others (e.g. laughing), compensation would mean that a conscious rule is developed: if someone makes a nonliteral statement and laughs, it is probably a joke. Otherwise it is probably a lie.

The following observations, which we describe in the next three sections, come from our clinical practice: Socio-cognitive tasks can be solved either intuitively-automatically or cognitively-deliberatively. The following example illustrates this: When a happy face is perceived, the intuitive automatic solution would be “the face shows happiness”. In the case of the cognitively-deliberative solution, different features are combined for interpretation, such as the cheek-raiser and the lip corner puller. This corresponds to the compensatory strategy used by autistic people which can be used to circumvent problems in the socio-cognitive area. However, it requires a great deal of effort on the part of the autistic person. The disadvantage of most of the experiments is that one can concentrate on the tasks and solve them in a cognitive-deliberative way.

Adults with ASD often learn to read the mental states of their fellow human beings via cognitive compensation when they are consciously thinking about them. Most experimental designs can be solved in this way. This could explain the results showing no significant difference in speech interpretation between the ASD and NTC groups.

For neurotypical people, the construction of a ToM often occurs unconsciously, i.e. when they are not thinking about it. An example would be the perception of mental states of hearers during a speaker’s lecture. In our clinical experience, this is not the case for people with ASD, as their focus needs to shift to consciously inferring the mental states of others.

In the research on disfluencies in speech two types of pauses are often discussed: silent pauses and filled pauses [cf. (86): 49; see also (87)]. As Rose [ (86): 49] points out, silent pauses are periods of non-articulation by the speaker, whereas filled pauses are periods of articulation of non-propositional content and also conform to language-specific conventions. Filled pauses are also often referred to as hesitations [for a discussion of the variation in terminology of filled pauses see Belz, (88): 1].

Silent and filled pauses have in common that they are used for speech planning and self-repair [cf. Rose, (86): 49]. Silent pauses are used for breathing and for marking syntactic structures, whereas filled pauses are periods of articulation of non-propositional content [cf. (86): 49] and are relevant for turn holding [see (89)].^^6^^

According to Belz [ (91): 41], filled pauses may serve as hesitation markers, repair markers, turn holding markers and others. The work of Wehrle et al. (92) with adults with ASD without intellectual impairment showed that a higher proportion of filled pause tokens were produced with the canonical level pitch contour by the NTC group compared to the autistic speakers.

The pragmatic difference between silent and filled pauses is less relevant for us because the right to speak does not play a role in our scenario. Nevertheless, we test whether filled and silent pauses differ in terms of the attribution of uncertainty. We use a combination of silent and filled pauses to realize particularly long and conspicuous hesitations.

At the phonetic level, the study by Betz et al. (93) suggested that the position of the extension in noun phrases such as ‘the green tree’ influences uncertainty perception. The results showed the following: Firstly, hearers interpreted lengthening in the initial position of a word as uncertainty about the semantic domain represented by the word itself. Secondly, hearers interpreted lengthening in the final position within the word as uncertainty about the semantic domain represented by the following content word [cf. (93): 3993]. As we used only one-word utterances in our study (un)certainty must be ascribed by the hearer to the information conveyed by this word.

Termis like hesitation and (dis)fluency are used differently in the literature [see (94)]. In our study, we use the term *hesitation* to refer to particles like “uh” which we also refer to as *fillers*. *Hesitation* and *pause* are each defined as independent variables for optimal manipulation of the synthetic signal [see (**Table 1**)]. However, we are aware that the hesitation particle and pause often form a unit in spoken utterances.

**Table 1:** Nine different combinations of the three cues pause, hesitation and intonation.

Pause	Hesitation	Intonation	Level
–	–	–	Certainty (Cer)
–	+	–	Hesitation (Hes)
+	–	–	Pause (Pau)
–	–	+	Intonation 1 (Into1)
–	–	+	Intonation 2 (Into2)
+	+	–	HesPau
–	+	+	HesInto2
+	–	+	PauInto2
+	+	+	PauInto2Hes

In our study the aim was to investigate whether the hearer attributes uncertainty to the speaker solely on the basis of prosodic information. As already mentioned, we regard the attribution of uncertainty to another person, i.e. the speaker, as a case of affective ToM, but with reference to conceptual content (a statement or a fact which the speaker is uncertain about). It is important to note that in our scenario the speech signal is synthetic, as we expressed different degrees of intended uncertainty through prosody using articulatory speech synthesis (33). The uncertain synthetic utterance served as an answer in the form of a statement to a question in a brief human-machine scenario. We will refer to previous studies in which uncertainty was modeled using a speech synthesizer.

### 1.2 Modelling and perception of uncertainty in human-machine-communication

In the context of human-machine interaction, the question arises as to whether speech synthesis should be enriched with emotional expressions [for a recent discussion of the role of emotions in synthetic speech see (95)]. According to Murray and Arnott (96), one aspect of the naturalness of the synthetic utterance is that the emotional state of the speaker contributes to the variability of synthetic speech; emotional expressions are regarded as pragmatic variations in speech. Artificial question-answering systems may follow in order to maintain user trust by expressing the degree of uncertainty attached to the provided answers (97). According to Székely et al. [ (98): 804], the expression and communication of a system’s internal uncertainty is a key to successful human-robot interaction.^^7^^

In previous studies, disfluent speech for acoustic speech synthesis has been modeled using filled pauses (99) and also of filled pauses and lexical fillers (100) in unit selection speech synthesis.^8^ In both studies, the activation of hesitations was not perceived differently with respect to naturalness from deactivation. Hönemann and Wagner (102) modeled uncertainty in speech synthesis as one of four emotional states by using features of prosody and voice quality. Furthermore, in the study of Śzekely et al. (98) the perception of uncertainty in synthetic speech was tested by using a synthesis method based on a DNN (deep neural network). Decreased vocal effort, filled pauses and prolongation of function words contributed to an increase in the degree of perceived uncertainty. For an overview of the role of hesitations in spoken dialogue systems, see Betz (103).

In traditional approaches for speech synthesis evaluation [e.g. (104)], the quality of synthetic speech was assessed, among other measures, by hearers’ judgments. Typically, hearers were asked to rate the naturalness and comprehensibility of the synthetic speech [cf. (104): 1012].

In our work, we used the concept of measuring naturalness and comprehensibility to evaluate the synthetic utterances. It should be noted that Wagner et al. (105) discussed the current state of the art in TTS evaluation and presented a new research program for speech synthesis evaluation in a paper published after we had collected the data for this study. The authors suggested that contextual appropriateness plays a crucial role in speech synthesis evaluation. They argued that the specific application and listening situation needs to be taken into account [cf. (105): 105].

For our research goal, however, we were interested in testing whether the articulatory synthetic utterances were perceived as natural. Our aim was not to evaluate the synthetic utterances, but to perceptually test whether the utterances were natural and understandable, in order to rule out that these dimensions function as confounding variables. Furthermore, the purpose of the fictive machine application in our experimental scenario remains too vague to assess contextual appropriateness.

In our previous work on uncertainty perception (106–108) different degrees of intended uncertainty were modeled with articulatory speech synthesis (33) and tested whether neurotypical adult hearers were able to discriminate between the degrees of uncertainty. The synthetic answers were part of a human-machine scenario in which the question was spoken by a human and the answer was the synthetic utterance. The acoustic cues rising intonation, pause and hesitation particle (“uh”) were systematically varied in Lasarcyk et al. (106) and in Wollermann et al. (107). Students from the University of Duisburg-Essen, as neurotypical hearers, were asked to judge the synthetic answers in terms of uncertainty and naturalness.^9^ In both works an additive principle of the uncertainty cues was described, i.e. the combination of two cues led to a higher level of perceived uncertainty than single cues. The study by Lasarcyk et al. (106) showed no significant difference between judgments when comparing the relative contribution of the single cues *intonation* vs. *filler*. Similarly, in Wollermann et al. (107), the single cues *pause* vs. *filler* were not rated significantly differently in terms of perceived uncertainty, but *intonation* was rated significantly more strongly regarding uncertainty than *pause*. Both Lasarcyk et al. (106) and Wollermann et al. (107) found no correlation between the ratings of uncertainty and the naturalness of the stimuli.

The material used in our pilot study (109) was based on the material of our previous studies (106, 107). In the following, when we refer to our pilot study we mean the study described by Bellinghausen et al. (109). However, we created new articulatory speech utterances with the revised version of Vocal Tract Lab (33) conveying different degrees of uncertainty. The answer to each question was generated by varying *pause*, *intonation*, and *hesitation* as acoustic cues. In the perception task, 28 neurotypical student hearers rated each answer on a rating scale in terms of uncertainty, naturalness and comprehensibility. The results indicated different contributions of acoustic cues to uncertainty perception. The effect of *intonation* and *hesitation* was more evident than the effect of *pause*. We observed an additive principle of the three cues, i.e. the more cues of intended uncertainty were activated, the higher was the perceived degree of uncertainty. The implications can be summarized as follows: In our study, we were able to model different degrees of intended uncertainty using articulatory speech synthesis by different combinations of pause, hesitation and intonation. Neurotypical adult hearers, i.e. students from the University of Duisburg-Essen, were generally able to discriminate the different levels in perception, although the relative contribution of the acoustic cues varied.

## 2 Method

In the current study, we aim to apply our experimental paradigm for measuring prosodic uncertainty in neurotypical hearers in our pilot study (109) to the investigation of prosody perception in autistic adult hearers. Thus, this study presents a feasibility study. We will present acoustic cues of uncertainty generated by articulatory speech synthesis to autistic adult listeners. To incorporate the developmental perspective, future work could modify the method to test autistic children and adolescents (see the Discussion).

### 2.1 Goal and research question

Our central research question was the following: Is there a group difference in the perception of uncertainty between hearers without and with ASD? We assumed that the prosodic marking of uncertainty in the speech signal has an effect on the perception on the side of the hearer. As mentioned above, we consider the attribution of uncertainty as part of the affective ToM with respect to a propositional content, here the answer given in a short question-answer scenario. Furthermore, we hypothesized that the marking of uncertainty is less dependent on the structure and semantics of the utterance than other prosodic phenomena such as focus [for an empirical investigation of focus theories see (30); 51]. Therefore, there is less interaction with syntactic and semantic processing and the information conveyed by prosody can hardly be induced by other linguistic information.

With our study we hope to contribute to the understanding of prosodic processing in autistic adult hearers by focusing on uncertainty as an emotional expression.

### 2.2 Hypotheses

Our primary hypothesis was as follows: There are significant differences in the perception of uncertainty between the ASD group and the NTC group. A low level of expressed intended uncertainty would be perceived as less uncertain by the ASD group than by the NTC group.

The secondary hypothesis was based on the results of our previous studies (106, 107) and was as follows: There would be a monotonic direct relationship between the number of prosodic uncertainty cues and participants’ ratings of uncertainty, regardless of group membership.

We used naturalness and intelligibility as quality measures for speech synthesis to see to what extent differences in naturalness (perception) can act as confounding variables. In our previous studies (106, 107) we only measured naturalness as a standard method for evaluating uncertain synthetic speech. In the current work, we include both naturalness and intelligibility as possible confounding variables.

The quality of speech synthesis may vary under different conditions. We include these two factors in addition to uncertainty in the listeners’ evaluation.

#### 2.2.1 Material

We use the material that we have already tested in our pilot study (109). To express different intended levels of uncertainty, utterances generated by the articulatory speech synthesizer (33) were used. This allowed us to manipulate specific prosodic parameters while minimizing the influence of unintended variation compared to natural speech.^10^

We chose the articulatory speech synthesizer VocalTractLab 2.2 by Birkholz (33) to generate high quality speech sounds while manipulating the parameters of the time-varying laryngeal and supra-laryngeal actions [cf. (109): 39]. The synthesizer has several components. To simulate the articulation process, 23 parameters control the geometric 3D model of a male vocal tract (110). A self-oscillating model of the vocal folds (36) is controlled by six parameters to specify the following features: subglottal pressure, fundamental frequency, and the rest shape of the glottis. The movements of the models of the vocal tract and the vocal folds are controlled by a gestural score. In this way, it is possible to manually adjust the movements for each word and to use different prosodic features for speech generation [cf. (109): 39].

In contrast to the articulatory speech synthesizer used here, state-of-the-art unit selection or neural synthesizers usually do not allow the individual manipulation of prosodic parameters such as f0 without causing involuntary changes in other prosodic parameters (e.g. voice quality) or articulation at the same time. This would make the specific assessment of the perceptual effect of individual prosodic parameters unreliable. Another way to manipulate prosodic parameters would have been to use a voice morphing method such as the change gender function in Praat (111), but this may introduce small acoustic artefacts in the manipulated signal, depending on the properties of the original signal (e.g. the irregularity of the voice).

The synthetic utterances were part of short question-answer pairs embedded in a human-machine interaction scenario designed to motivate the use of synthetic speech. The scenario was presented to the participants as follows: The question in German language was spoken by a natural voice (*Was siehst Du?/What do you see*)*?* and asked by a research assistant who showed pictures of fruit and vegetable objects to an image recognition robot. The synthetic answer, such as *Bananen/Bananas*, was given by the robot. The robot recognized the items with a certain level of confidence and was able to express uncertainty about the recognition in its answer. The critical stimuli were the following trisyllabic one word sentences in German: *Bananen/bananas, Limetten/limes, Melonen/melons, Tomaten/tomatoes* [cf. (109) 40]. We have opted for one-word sentences because they represent the smallest meaningful unit for an answer. In total, there were nine different levels of intended uncertainty, i.e. all possible combinations of the three cues *pause*, *hesitation* and *intonation* [see (**Table 1**)]. In addition, the following one-word phrases were used as distractors (without uncertainty cues) to the synthetic speech signal in order to minimize learning effects when the recipients judged the critical stimuli: *Birnen/pears, Blaubeeren/blueberries, Bohnen/beans, Erdbeeren/strawberries, Gurken/cucumbers, Knoblauch/garlic, Mandarinen/mandarins, Orangen/oranges and Paprika/paprica* [cf. (109) 40].

Following Bellinghausen et al. [ (109): 40], we describe below the three cues *pause*, *hesitation* and *intonation* used to generate the experimental stimuli.

Pause: This cue refers to the time between the question and the answer. For each level of intended uncertainty, a default silent pause of 1 s was used between the question and the answer. When the pause was activated (pause[+]), we used either a silent pause of 4 s as strongly marked pause or a filled pause,^11^ i.e. the hesitation äh/uh with a duration of 0.37 followed by a silent pause of 3.632 s giving a total duration of 4 s [cf. (109): 40].

It has to be noted that the pause can have other functions than expressing uncertainty. In this scenario, it could also be interpreted as the robot’s processing time while producing the synthetic utterance. In our previous study (109) it emerged from the text comments that the robot was obviously considered to be uncertain. However, due to the close relationship between uncertainty and processing time, these two aspects cannot be separated.

Hesitation: The hesitation particle *äh/uh* was either present (hes[+]) or absent (hes[-]) [cf. (109) 40].

**Figure 1:**
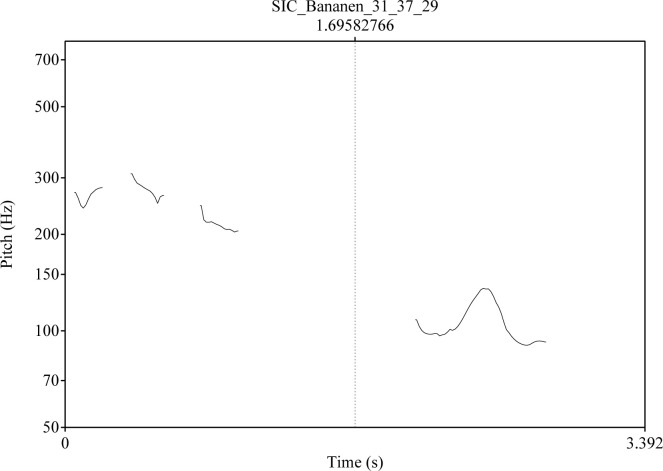
Intonation contour for the question “Was siehst Du/What do you see?” (left side) and for the answer Bananen/Bananas; level: Certainty (Cer) [see also (109): 41].

**Figure 2:**
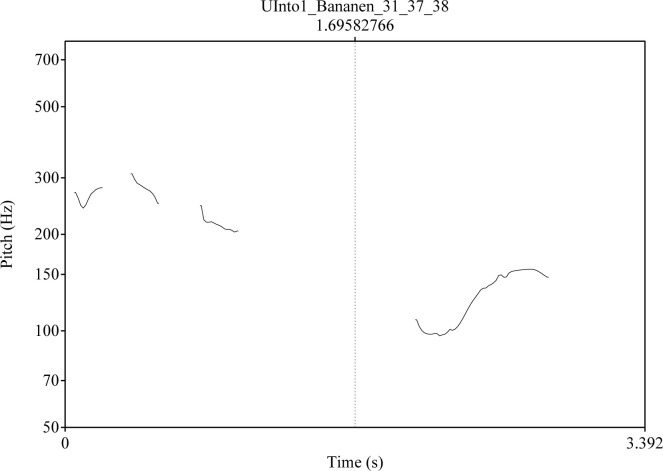
Intonation contour for the question “Was siehst Du/What do you see?” (left side) and for the answer Bananen/Bananas; level: Intonation 1 (int) [see also (109): 41].

**Figure 3:**
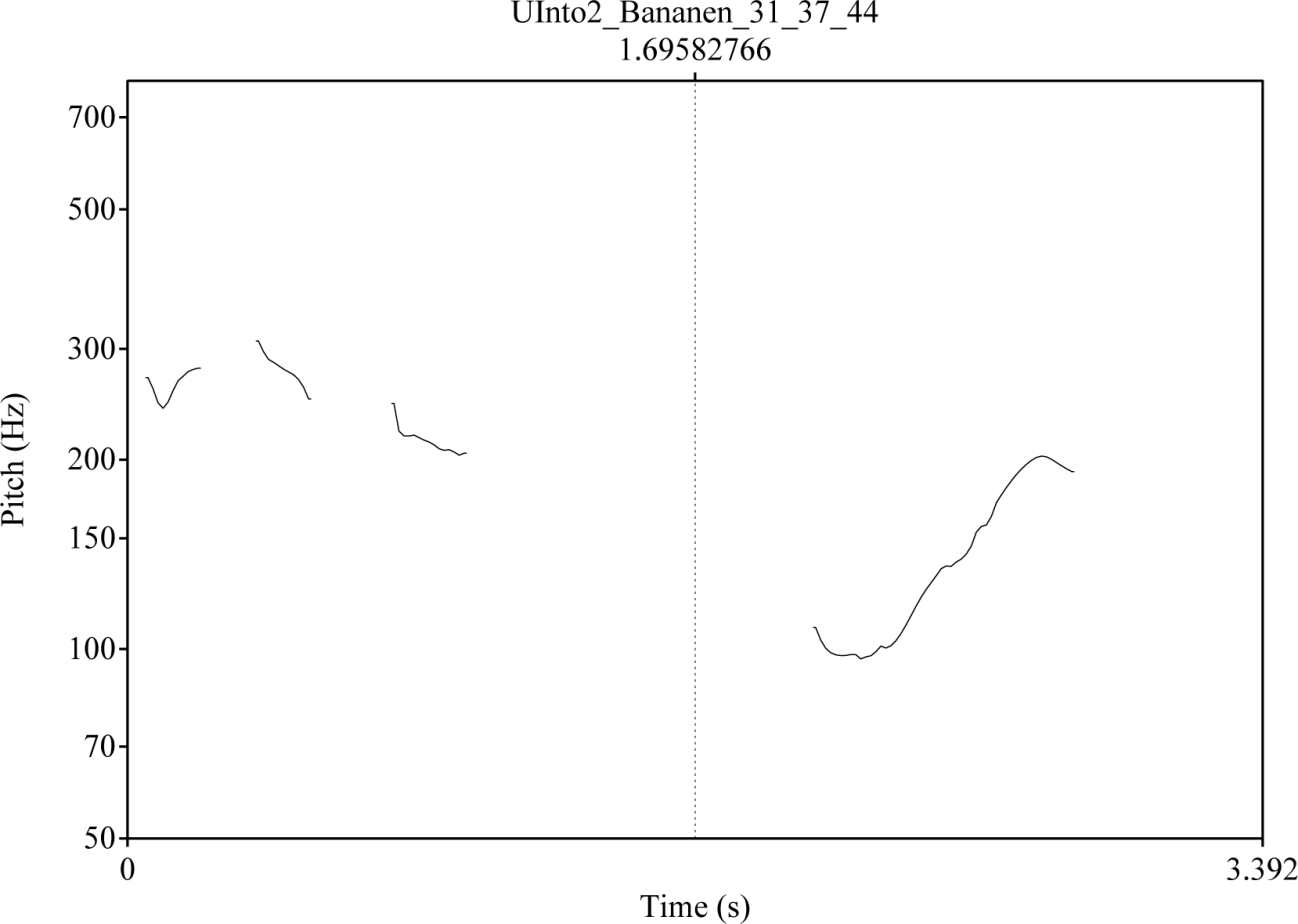
Intonation contour for the question “Was siehst Du/What do you see?” (left side) and for the answer Bananen/Bananas; three intended levels of uncertainty:; level: Intonation 2 (Into2) [see also (109): 41].

Intonation: The intended level of certainty was expressed by a falling contour with a difference of 8 ST (semitones) between the highest pitch on the stressed syllable of the word and the lowest pitch at the end of the utterance. In addition, two intonation contours were used to express intended uncertainty. In the level *Into1*, the pitch of the last syllable rises by 8 ST (semitones) above the lowest pitch in the first syllable for moderate uncertainty, and in *Into2* it rises by 13 ST for intended strong uncertainty [see also (109) 40]. Different intonation contours for the critical stimulus *Bananen* are shown in **Figures 1**–**3**. The pitch contour on the left side is the question uttered by a human speaker. On the right the pitch contour of the synthetic answer is shown.

The number of critical stimuli was 36 (4 one-word utterances x 9 conditions). There were also 9 distractors and one practice trial *Was siehst Du?/What do you see?* The stimulus *Rosinen/Raisins* was presented at the beginning of the experiment. In order to minimize the influence of participants’ learning effects on their perceptual judgments, we constructed four task sets. Each critical item occurred only once within the four sets. Thus, each task set consisted of the practice trial, nine critical items complemented by nine distractors; the order of presentation of critical trials and distractors was randomized in advance. In this way, each participant had to work on one task set of 19 trials with question-answer pairs. Within each group, the four task sets were counterbalanced across participants (see Appendix for the experimental design). As we wanted to use as many potentially relevant questionnaires and control tests as possible in the feasibility study, we had to limit the number of trials in the prosody test to n=1 per condition, so that the experimental session would not be too long and become too strenuous, especially with regard to our patients.

#### 2.2.2 Participants

56 participants (age range: 18-65, IQ > 80) with German as their first language took part in the study. The ASD group consisted of 28 adults (12 female, 16 male) diagnosed according to ICD-10 criteria (F84.0 Childhood autism, F84.1 Atypical autism, F84.5 Asperger syndrome). Only for the ASD group the ADOS-2, Module 4 was used (scale Communication + Social Interaction Total: *M*=8.04, *SD*=4.46). There were also 28 neurotypical adults (14 female) in the NTC. As shown in **Table 2**, there were no significant group differences in terms of age, gender, and IQ. In terms of autistic symptomatology, the ASD group had significantly higher values on the two self-report measures SRS-2 Adult Self-Report (ASD: *M*=112.50, *SD*=28.50; NTC: *M*=33.89, *SD*=19.07; *t*_(54)_= 12.13, *p* <.001) and the AQ [ASD: *M*=38.61, *SD*=7.16; NTC: *M*=13.29, *SD*=6.42; *t*_(54)_= 13.93, *p* <.001)].

**Table 2:** Sample characteristics: Age, gender, IQ, and autistic symptomatology for the ASD and the NTC group.

	ASD			NTC			Test statistic	*p*
	*n*	*M*	*SD*	*n*	*M*	*SD*		
**Age**	28	44.68	11.68	28	41.61	14.04	*t*_(54)_ = 0.89	.378
IQ								
CFT 20-R	28	111.36	21.68	28	108.61	12.92	*t*_(54)_ = 0.58	.567
MWT-B	28	113.79	15.65	28	117.32	14.34	*t*_(54)_= -0.88	.382
**SRS-2 Adult Self-Report**	28	112.50	28.50	28	33.89	19.07	*t*_(54)_= 12.13	<.001
**AQ**	28	38.61	7.16	28	13.29	6.42	*t*_(54)_= 13.93	<.001
**ADOS-2 Module 4**	28	8.04	4.46					

ASD, Autism Spectrum Disorder; NTC, Neurotypical Controls.

Gender ratio (m:f): ASD: 12: 16; NTC: 14: 14;χ2(1) = 0.287, p = .592.

Participants were screened for eligibility with regard to inclusion and exclusion criteria prior to the study. Exclusion criteria for the study participants were an IQ < 80, non-native speaker of German, as well as an acute depressive episode, psychotic symptoms or suicidal tendencies.

Regarding the language abilities of the autistic participants, it is noted that they completed the CFT 20-R and MWT-B test [see (**Table 2**)].The Basic Intelligence Test (CFT) is considered culturally fair because it is based on non-verbal and illustrative test tasks. It measures basic mental ability (g-factor) independent of socio-cultural and educational influences. The CFT 20-R consists of two similarly structured test parts with the four subtests: Series Continuation, Classification, Matrices, and four Topological Conclusions. The Multiple Choice Vocabulary Intelligence Test (MWT-B) measures general vocabulary. Intelligence levels. For each item, the candidate has to find the correct German word from five given words and four nonsense words.

The study took place at the Department of Psychiatry and Psychotherapy of the Medical Center - University of Freiburg, Germany. Participants with ASD were recruited through the outpatient clinic or from inpatient wards or after their discharge, and through the website and notices of the autism outpatient clinic.

#### 2.2.3 Procedure

The following instruments [see (**Table 3**)] were performed as part of the study: both the ASD and the NTC group completed self-report questionnaires on the AQ, EQ, SRS-2 Self-Report, BDI-II, BVAQ and FQLP prior to the examination. Furthermore, the SCL-90-S was administered to the NTC group only. Interviews about the psychotic symptoms and two IQ tests, the CFT 20-R and the MWT-B, were also administered to both groups before the examination. In addition, the diagnosis of the ASD group was confirmed by the ADOS-2.

**Table 3:** Instruments and test procedures used.

	Groups	
	ASD	NTC
IQ (CFT 20-R & MWT-B)	x	x
Prosody test	x	x
Audiometric test	x	x
Minimum Pitch Discrimination test	x	x
Minimum Pitch Change test	x	x
Recording of medications	x	x
ADOS-2	x	
Interview on psychotic symptoms	x	x
AQ (self-report)	x	x
EQ (self-report)	x	x
SRS-2 (self-report)	x	x
BDI-II (self-report)	x	x
BVAQ (self-report)	x	x
Freiburg Language Pragmatics Questionnaire (self-report)	x	x
SCL-90 (self-report)		x

ASD, Autism Spectrum Disorder; NTC, Neurotypical Controls.

The AQ, EQ, SRS-2 self-report and FQLP questionnaires were used to characterize autistic symptoms. The BVAQ was collected because of possible alexithymic symptoms, which are more common in ASS. The ADOS was only collected from the ASS group in order to describe the communicative and social-interactive behavior of this group. The SCL-90 was only used in the NTC group to detect signs of psychiatric disorders.

Participants were informed of the aim and the procedure of the study, and a short interview was conducted to exclude possible psychotic symptoms for the participants with ASD. All participants signed an informed consent. The study was approved by the ethics committee (EK-Freiburg: 558/17). It was conducted in accordance with the Declaration of Helsinki. The experimental session included a prosody test, a complementary audiometry test, a pitch discrimination task and a pitch change task assessing sensory pitch perception. Data were collected during a two-hour individual session with the participants.

##### 2.2.3.1 Prosody test

In the prosody test, participants were presented with short question-answer pairs consisting of the natural language question *Was siehst Du?/What do you see?* and the articulatory synthetic utterance serving as an answer, e.g. *Bananen/bananas*. The synthetic response instantiated one of the nine experimental conditions in which the three cues *intonation*, *pause*, and *hesitation* were either present or absent [see (**Table 1**)].

The prosody test was presented to the participants via a computer program (see Appendix for the experimental design). Each participant completed 19 trials (nine levels of intended uncertainty plus nine distractors following an example stimulus). Each question-answer pair was played only once. Participants were asked to rate a) uncertainty b) naturalness, and c) comprehensibility of the synthetic response on a 5-point rating scale (1 = *uncertain/little natural/little comprehensible* and 5 = *certain/very natural/very comprehensible*). In contrast to Bellinghausen et al. (109), the reaction time was also measured when rating the response.

As discussed in the introduction, we measure not only the perception of certain prosodic features in terms of perceived uncertainty, but also their effect on naturalness and comprehensibility.

##### 2.2.3.2 Audiometry

An audiometry test from Electronica-Technologies was used to ensure that the prosodic stimuli used were reliably recognized by the participants, and that they had no significant hearing loss. Each ear was tested separately. Sine tones (250 Hz, 500 Hz, 1000 Hz, 2000 Hz, 3000 Hz, 4000 Hz, 8000 Hz) were presented at increasing loudness via headphones.

##### 2.2.3.3 Minimal pitch discrimination and change

Following Globerson et al. (29), minimal pitch discrimination was used to investigate whether two sine tones of only slightly different frequency could be perceived as different. Thus, the level of the minimal perceived tone difference could have a significant influence on the perception of prosodic intonation [see also (29)]. The difference between the two tones amounted to 200 Hz at the beginning and was reduced to the minimum pitch difference perceived by the participant. Thus, if hearers can only perceive large differences between the reference and the comparison tone, this could have a significant impact on the perception of prosodic intonation [see also (29)].

The minimum pitch change detection for each participant was determined by assessing the course of a tone rising or falling in frequency. The test started with tone movements of 12 Hz up or down from the starting tone of 200 Hz. For reduction of the pitch change the same staircase function as for the pitch discrimination task was used according to Globerson et al. (29).

By testing pitch discrimination and pitch change detection, we wanted to ensure that basic auditory perception is not impaired in hearers with ASD without II and thus could be excluded from influencing prosody perception. Therefore, both pitch tests served as a kind of control condition in order to rule out the possibility that putative group differences could be explained by differences in mere low-level auditory processing.

#### 2.2.4 Statistical analysis

For the sample characteristics, group differences in age and IQ were tested using t-tests, and group differences in gender were tested applying the chi-square test. Since significant deviations from normality could be expected for all other variables (minimum pitch discrimination and change, ratings of uncertainty, naturalness and comprehensibility, and their corresponding response time variables), we conducted robust tests as described and recommended by Field and Wilcox (112), Mair and Wilcox (113) and Wilcox (114).

Robust methods address two key properties of a statistical test: the probability of a false positive, also known as a Type I error, and power, the probability of detecting true differences between groups (or a true association between two or more variables). They attempt to overcome serious drawbacks when assumptions of conventional methods such as ANOVA are violated, in order to avoid misleading results and interpretations [see (115) for more details]. To our knowledge, robust methods do not differ from classical non-parametric techniques (such as the Wilcoxon-Mann-Whitney test) in terms of controlling for item and individual variability.

For the analyses of minimum pitch discrimination and change, we had a one-factorial design with “diagnostic group” (ASD, NTC) as an independent factor. For the ratings of uncertainty, naturalness and comprehensibility, and their corresponding response time variables, we used a 2 x 8 design with the independent factor “diagnostic group” (ASD, NTC) and “prosodic condition” (Cer, Hes, Pau, Into2, HesPau, HesInto2, PauInto2, PauInto2Hes; see **Table 1** for a description of these conditions) as the dependent factor. For some analyses, we also considered the distractor trials as an additional prosodic condition and Into1 as a “milder” condition for an intonation (see above) that was not combined with the other two cues *pause* and *hesitation.* Therefore, only Into1 was statistically tested against Into2.

The 2 x 8 design was analyzed with a two-way mixed design robust test statistic [*bwtrim*, F-like test values, see (112): 29-30; (113): 479]. *t1waybt* is a robust one-way alternative with an outcome of F-like values for between-subjects effects and effect sizes [see (112): 28-29]. *yuend* is used as a robust alternative for a dependent t-test that also outputs the explanatory measure of effect size ξ. Similar to Pearson correlations, ξ = .10,.30, and.50 correspond to small, medium, and large effect sizes, respectively [ (112: 25-26; (113): 458] [see also (114): 506-511 for three factor design, 2 x 2 x 8].

All robust tests were performed with the same following parameters (except *bwtrim* without bootstrapping): trimmed mean with 20% trimmed scores (tr = 0.2), the modified one-step estimator (est = “mom”), and the number of bootstrapping samples of 5000 (nboot = 5000). In order to control the overall probability of a Type I error (false positive) for multiple hypothesis tests, *post-hoc* tests are reported after Bonferroni adjustment.

All statistical analyses were performed with R version 4.1.2 using the R package WRS2 version 1.1-3 with its collection of robust statistical methods. A significance level of α = .05 was used for hypothesis testing.

## 3 Results

### 3.1 Audiometry test

All participants in the study had unaffected hearing abilities at the frequencies measured.

### 3.2 Minimal pitch discrimination and change

There were no significant differences between the ASD and NTC groups in either pitch discrimination or pitch change perception or reaction time [see (**Table 4**)]. However, the ASD group descriptively achieved lower values for pitch discrimination and change (in Hertz) than the NTC. There was only a minimally longer response time for pitch change detection in the ASD group than in the NTC. No significant differences were observed.

**Table 4:** Test results for pitch variation and for pitch change. Minimum pitch discrimination in Hertz and reaction times in milliseconds.

	ASD			NTC			*Test Stat^a^*	*p^a^*	*ES^a^*
	*n*	*M*	*SD*	*n*	*M*	*SD*			
Pitch (in Hz)									
Discrimination	27	43.102	57.861	28	58.321	81.071	0.193	.668	0.101
Change	26	3.092	3.830	27	3.520	4.330	0.019	.900	0.026
RT (in ms)									
Discrimination	27	2469	1700	28	2472	1069	1.210	.280	0.201
Change	26	1241	842	27	1201	599	0.465	.510	0.140

ASD, Autism Spectrum Disorder; NTC, Neurotypical Controls.

Reasons for missing data: In the ASD group two participants dropped out before the end of the pitch tasks (two for the pitch change task and one for the pitch discrimination task); for one participant of the NTC group saving of the data failed for the pitch discrimination task.

a Values for Test Stat, p and ES (explanatory measure of effect size) are from robust ANOVA.

### 3.3 Prosody test

#### 3.3.1 Perception of uncertainty

##### 3.3.1.1 Distractor analysis

Before describing the results for the ratings of the critical stimuli in terms of perceived uncertainty, naturalness, and comprehensibility we report on the ratings of the distractor items. As mentioned above, we used 10 distractor items, all of which were exclusively generated in an intended certain way of speaking. As shown in **Table 5**, there was no significant difference between the ratings of uncertainty for the distractor trial condition Dist and the prosodic uncertainty condition Cer (*M* = 4.20, *SD* = 0.65 vs. *M* = 4.20, *SD* = 0.97; robust test statistic = -0.71, *p* = .482) for the whole sample, and also the pattern of the results of these two with all other conditions is remarkably similar [see (**Table 5**)]. This was also true for the ratings of uncertainty within the ASD group (Dist: *M* = 4.31, *SD* = 0.63, Cer: *M* = 4.21, *SD* = 0.92; robust test statistic = -0.18, *p* = .861) as well as in the NTC group (Dist: *M* = 4.15, *SD* = 0.67, Cer: *M* = 4.14, *SD* = 1.04; robust test statistic = -0.91, *p* = .375).

**Table 5:** Pairwise comparisons of ratings of uncertainty between prosodic uncertainty conditions (independent of diagnostic group).

Test stat^a^ *p* ^b^ ES ξ	*M*	*SD*	1. Dist	2. Cer	3. Hes	4. Pau	5. Into1	6. Into2	7. HesPau	8. HesInto2	9. PauInto2
1. Dist	4.234	0.651	–	–	–	–	–	–	–	–	–
2. Cer	4.179	0.974	-0.711 .482 0.074	–	–	–	–	–	–	–	–
3. Hes	2.482	1.236	**10.684** **<.0001** **0.873**	**9.485** **<.0001** **0.790**	–	–	–	–	–	–	–
4. Pau	3.214	1.261	**6.067** **<.0001** **0.748**	**5.720** **<.0001** **0.505**	**-3.708** **.0008** **0.427**	–	–	–	–	–	–
5. Into1	3.339	1.225	**4.211** **.0002** **0.607**	**4.331** **.0001** **0.589**	-3.153 .003 0.528	-0.591 .558 0.070	–	–	–	–	–
6. Into2	2.732	1.408	**6.773** **<.0001** **0.715**	**7.555** **<.0001** **0.674**	-0.951 .348 0.109	1.769 .086 0.279	2.420 .021 0.284	–	–	–	–
7. HesPau	2.143	1.212	**12.112** **<.0001** **0.755**	**12.232** **<.0001** **0.766**	2.499 .018 0.238	**6.277** **<.0001** **0.498**	**4.445** **.0001** **0.573**	2.625 .013 0.281	–	–	–
8. HesInto2	1.911	1.133	**12.660** **<.0001** **0.868**	**13.767** **<.0001** **0.818**	3.353 .002 0.378	**5.582** **<.0001** **0.598**	**5.859** **<.0001** **0.671**	**4.624** **<.0001** **0.522**	1.130 .267 0.143	–	–
9. PauInto2	2.018	1.258	**12.710** **<.0001** **0.855**	**13.025** **<.0001** **0.805**	3.096 .004 0.364	**6.192** **<.0001** **0.5**74	**6.078** **<.0001** **0.646**	**4.071** **.0003** **0.369**	1.105 .277 0.128	-0.150 .881 0.016	–
10. PauHesInto2	1.589	0.910	**20.333** **<.0001** **0.935**	**19.727** **<.0001** **0.891**	**5.639** **<.0001** **0.556**	**7.822** **<.0001** **0.712**	**7.812** **<.0001** **0.780**	**6.621** **<.0001** **0.696**	2,995 .005 0.334	2.436 .020 0.197	2.350 .025 0.214

Test stat, Test statistic: if sign is positive then row condition has lower ratings of uncertainty than column condition and vice versa; ES ξ, explanatory measure of effect size: Analogous to Pearson correlations, ξ = .10, .30, and .50 correspond to small, medium, and large effect sizes.

a n = 56 and df = 33 for all pairwise contrasts.

b The significance cutoff via Bonferroni correction is about p_cutoff_ = .05/45 = .00111. Significant differences are bold.

With respect to response time for the ratings of uncertainty, participants needed more time for the distractor trials than for the utterances in the condition Cer (*M* = 4511, *SD* = 2365 vs. *M* = 4230, *SD* = 4508; robust test statistic = 2.80, *p* = .008, ES = 0.27). This difference was also significant within the ASD group (Dist: *M* = 4966, *SD* = 2689; Cer: *M* = 4134, *SD* = 4866; robust test statistic = 2.95, *p* = .009, ES = 0.45), but not within the NTC group (Dist: *M* = 4056, *SD* = 1933; Cer: *M* = 4325, *SD* = 4208; robust test statistic = 0.95, *p* = .360, ES = 0.14). The distractors differ from the stimuli words in their syllable structure. These phonological discrepancies could explain the differences in reaction times.

##### 3.3.1.2 Ratings of uncertainty of the 2 x 8 design

**Figure 4:**
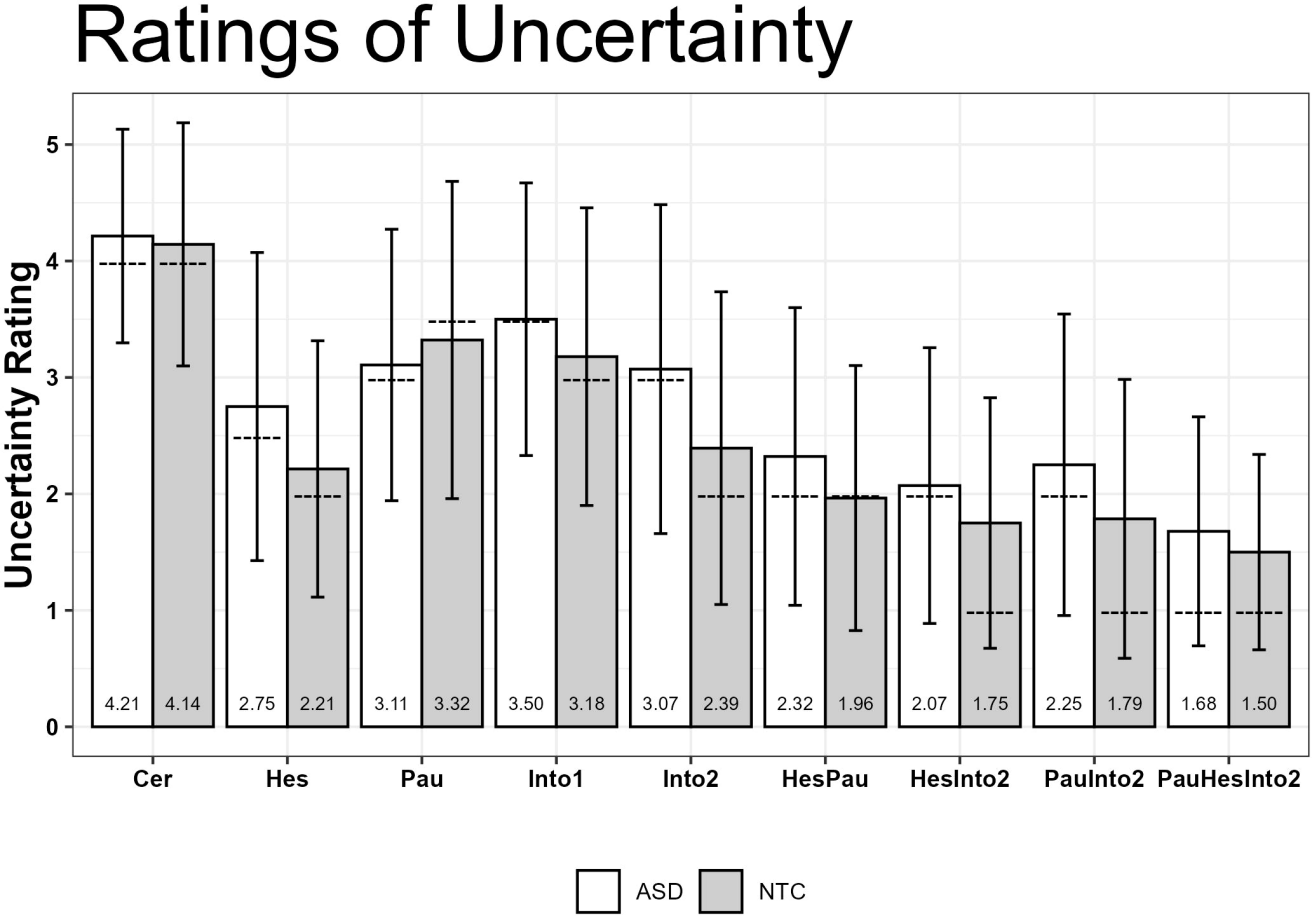
Means of uncertainty ratings (1=uncertain, 5=certain), dashed lines denote the median. Abbreviations for the prosodic uncertainty conditions are explained in **Table 1**.

In the statistical analysis of the ratings of uncertainty with robust ANOVA, the main effect of diagnostic group was not significant (robust test statistic *F*(1, 32) = 2.10, *p* = .160), whereas the main effect of prosodic uncertainty conditions was significant (robust test statistic *F*(7, 25) = 43.20, *p* <.0001). However, the interaction between these two factors was far from being significant (robust test statistic *F*(7, 25) = 1.30, *p* = .27). In **Figure 4**, means of the ratings of uncertainty for all factorial combinations are shown. Due to the non-significant interaction, *post-hoc* comparisons are only reported for the different levels of the significant condition main effect and for the hypothesized group main effect, but not for the non-significant interaction.

In **Table 5**, all pairwise comparisons between the prosodic uncertainty conditions (including the distractor trials) are reported. There are noteworthy differences between the condition CER (and, as already mentioned above, the distractors DIST) and all the other prosody conditions. Also, most of the 2-cue prosody conditions (HesPau, HesInto2, PauInto2) were judged to be more uncertain than the 1-cue prosody conditions (Hes, Pau, Into1, Into2). The 3-cue prosody condition PauInto2Hes which had descriptively the lowest mean, elicited significantly lower ratings of uncertainty than all the 1-cue prosody conditions, whereas differences to the 2-cue prosody conditions were not significant after Bonferroni correction. All significant differences between conditions had medium up to very large effect sizes (all ξs >.37).

The top part of **Table 6** shows all the contrasts in ratings of uncertainty between the two groups ASD and NTC for each prosodic uncertainty condition. The corresponding means are shown in **Figure 4**. As we had nine different conditions and three combined comparisons the number of comparisons was twelve, and therefore our p-values should be below.05/12=.004166 in order to be considered as significant after Bonferroni correction. Descriptively, the ASD group had higher ratings of uncertainty in all conditions except for the condition Pau. However, the most pronounced between group difference for the combined condition “All cues”, was not significant after Bonferroni correction.

**Table 6:** Differences between ratings of uncertainty for ASD and NTC.

Ratings of Uncertainty	ASD (*n*=28)	NTC (*n*=28)	*Test Stat*	*p^a^*	ES ξ
	*M* (*SD*)	*M* (*SD*)			
Cer	4.214 (0.917)	4.143 (1.044)	0.068	.777	0.050
Hes	2.750 (1.323)	2.214 (1.101)	2.328	.136	0.308
Pau	3.107 (1.166)	3.321 (1.362)	0.760	.392	0.196
Into1	3.500 (1.171)	3.179 (1.278)	0.861	.358	0.177
Into2	3.071 (1.412)	2.393 (1.343)	2.662	.112	0.331
HesPau	2.321 (1.278)	1.964 (1.138)	0.836	.363	0.208
HesInto2	2.071 (1.184)	1.750 (1.076)	1.247	.266	0.212
PauInto2	2.250 (1.295)	1.786 (1.197)	2.660	.117	0.336
PauHesInto2	1.679 (0.983)	1.500 (0.839)	0.343	.501	0.156
MEAN					
Single cues	2.976 (0.884)	2.643 (0.934)	2.343	.127	0.297
Two combined cues	2.214 (1.019)	1.833 (0.918)	3.392	.077	0.340
All cues	2.464 (0.868)	2.133 (0.826)	4.133	.043	0.333
Uncertainty reaction time [ms]	ASD (*n*=28)	NTC (*n*=28)	*Test Stat*	*p^a^*	ES ξ
	*M* (*SD*)	*M* (*SD*)			
Cer	4134 (4866)	4325 (4208)	0.603	.441	0.138
Hes	5109 (3697)	3661 (2901)	3.149	.102	0.473
Pau	3504 (3076)	3342 (3138)	1.242	.264	0.221
Into1	5095 (4604)	3537 (1635)	0.098	.767	0.080
Into2	3986 (2293)	3667 (2384)	0.890	.341	0.200
HesPau	4759 (4238)	4409 (5932)	0.429	.520	0.137
HesInto2	4683 (2537)	2629 (1165)	9.880	.014	0.656
PauInto2	3469 (1909)	2992 (1318)	0.582	.466	0.160
PauHesInto2	4891 (5530)	2974 (1839)	2.781	.110	0.325
MEAN					
Single cues	4200 (2085)	3557 (1981)	2.900	.095	0.337
Two combined cues	4304 (2220)	3344 (2161)	4.361	.050	0.411
All cues	4343 (2104)	3382 (1698)	4.343	.044	0.398

ES, effect size; abbreviations for intended uncertainty levels are explained in **Table 1**. Means are calculated for single cues: Hes, Into, Pau; for two combined cues: HesPau, HesInto2, PauInto2; for all cues: eight single, combined and triple uncertainty cues together. Reaction times are listed in ms (milliseconds).

The significance cutoff via Bonferroni correction is approximately p_cutoff_ = .05/12 = .004166.

a Values for Test Stat, p and ES (explanatory measure of effect size) are from robust tests. ES ξ = explanatory measure of effect size: Analogous to Pearson’s correlations, ξ = .10, .30, and .50 correspond to small, medium, and large effect sizes. Mean values for uncertainty perception are shown in the subtable on top, values for reaction times in milliseconds are listed in the subtable below.

##### 3.3.1.3 Response times of ratings of uncertainty for the 2 x 8 design

In the statistical analysis of the response times of the ratings of uncertainty with robust ANOVA, the two main effects and the interaction were not significant (main effect “diagnostic group”: robust test statistic *F*(1, 30) = 3.40, *p* = .074; main effect “prosodic uncertainty condition”: *F*(7, 22) = 2.30, *p* = .061; interaction: *F*(7, 22) = 1.70, *p* = .163).

Descriptive statistics for all contrasts between the two groups ASD and NTC for each prosodic uncertainty condition are presented in the lower part of **Table 6**. As can be seen, the ASD group needed descriptively more time to reach ratings of uncertainty in almost all prosodic uncertainty conditions (except for the condition “Cer”). The largest difference can be seen in the condition HesInto2: The ASD group took almost twice as long (4683 ms) as the NTC group (2629 ms). Note that this difference is no longer significant after Bonferroni correction (robust test statistic = 9.90, *p* = .001, ES = 0.66).

When integrating the data on perceptual judgments for single cues, two combined cues, and all the cues, the following was observed: the mean for the ASD group was always higher compared to the NTC, i.e. the ASD group needed more time to rate the different levels of uncertainty than the NTC, but the differences were no longer significant after Bonferroni correction.

##### 3.3.1.4 Exploratory analyses: effect of gender, IQ, and severity of autistic symptoms on the processing of uncertainty cues

In order to assess whether or not other variables might influence the processing of uncertainty cues, we also conducted exploratory statistical analyses with the possible impact factors of gender, IQ, and degree of autistic symptom severity.

IQ. Concerning the IQ, we computed Spearman rank-order correlations (*r_s_*) for both IQ measures (CFT 20-R and MWT-B) with the ratings of uncertainty and also for the response times within each experimental uncertainty cue condition for the total sample and additionally also for the NTC and ASD groups separately. For the IQ measures, we found no significant Spearman rank-order correlations between IQ and ratings of uncertainty for the total sample (CFT 20-R: all *r_s_* in [-.167; +.160], all *p*s >.219; MWT-B: all *r_s_* in [-.139; +.092], all *p*s >.309) as well as for both groups (ASD: CFT 20-R: all *r_s_* in [-.288; +.366], all *p*s >.055; MWT-B: all *r_s_* in [-.202; +.067], all *p*s >.302; NTC: CFT 20-R: all *r_s_* in [-.157; +.369], all *p*s >.053; MWT-B: all *r_s_* in [-.125; +.259], all *p*s >.183).

There were no significant correlations for the response times of the ratings of uncertainty with the IQ measure CFT 20-R (Total: all *r_s_* in [-.199; +.145], all *p*s >.141; ASD: all *r_s_* in [-.364; +.100], all *p*s >.057; NTC: all *r_s_* in [-.325; +.350], all *p*s >.068). Similarly, for the MWT-B, almost all correlations were not significant except for two coefficients in the NTC group (Total: all *r_s_* in [-.083; +.156], all *p*s >.251; ASD: all *r_s_* in [-.326; +.166], all *p*s >.091; NTC: all *r_s_* in [+.060; +.459], *r_s_* = +.459, *p* = .014 in condition Hes and *r_s_* = +.459, *p* = .014 in condition PauInto2Hes, all other *p*s >.120). It should be noted that all mentioned p-values are uncorrected with respect to multiple testing.

Degree of autistic symptom severity. As the degree of autistic symptom severity is strongly associated with the diagnostic group membership (see **Table 2**), it is useful to check for correlations within diagnostic groups only in order to assess whether or not this variable has an additional influence on the processing characteristics of uncertainty cues. For the ratings of uncertainty with the autistic symptom severity measure SRS-2 Adult Self-Report there were no significant correlations except for the conditions Hes, Pau, and HesPau within the ASD group, and for the condition Cer within the NTC group (ASD: all *r_s_* in [-.314; +.627], *r_s_* = +.487, p = .009 in condition Hes, *r_s_* = +.627, *p* <.001 in condition Pau, *r_s_* = +.498, *p* = .007 in condition HesPau, all other *p*s >.100; NTC: all *r_s_* in [-.409; +.253], *r_s_* = -.409, *p* = .031 in condition Cer, all other *p*s >.063). For the autistic symptom severity measure AQ, there were no significant correlations except for the condition HesPau for the ASD group (ASD: all *r_s_* in [-.237; +.404], *r_s_* = +.404, *p* = .033 in condition HesPau, all other *p*s >.062; NTC: all *r_s_* in [-.258; +.261], all *p*s >.180). No significant correlations were found for the ADOS-2 (ASD: all *r_s_* in [-.280; +.276], all *p*s >.149).

There were no significant correlations for the response times of the ratings of uncertainty with the autistic symptom severity measure SRS-2 Adult Self-Report, except for one condition within the NTC group (ASD: all *r_s_* in [-.233; +.126], all *p*s >.233; NTC: all *r_s_* in [-.023; +.417], *r_s_* = +.417, *p* = .027 in condition Into1, all other *p*s >.167). No significant correlations were found for the autistic symptom severity measure AQ (ASD: all *r_s_* in [-.035; +.327], all *p*s >.090; NTC: all *r_s_* in [-.015; +.356], all *p*s >.063): For the ADOS-2, only one significant correlation with response times was found in the condition Cer (ASD: all *r_s_* in [+.080; +.438], *r_s_* = +.438, *p* = .021 in condition Cer, all other *p*s >.094).

Gender. In order to assess a potential influence of gender on the processing of uncertainty cues, we added gender as an additional independent factor in the robust ANOVA. There was no significant main effect of gender on ratings of uncertainty (*F*(1, 999) = 1.46, *p* = .228), nor were there any significant interactions of the other factors with gender (gender x diagnostic group: *F*(1, 999) < 1; gender x prosodic uncertainty condition: *F*(9, 999) < 1; gender x diagnostic group x prosodic uncertainty condition: *F*(9, 999) < 1). A similar pattern was found for response times: No significant main effect for gender (*F*(1, 999) = 2.30, *p* = .130) and no significant interactions of the other factors with gender (gender x diagnostic group: *F*(1, 999) < 1; gender x prosodic uncertainty condition: *F*(9, 999) < 1; gender x diagnostic group x prosodic uncertainty condition: *F*(9, 999) < 1).

In summary, the exploratory analyses revealed no strong evidence that gender or IQ are reliably related to the processing of prosodic uncertainty cues. There was weak evidence that severity of autistic symptoms may play an additional role beyond mere diagnostic group membership.

#### 3.3.2 Perception of naturalness and comprehensibility

In section 2.2, the (perceived) quality of the synthetic stimuli was mentioned as a possible confounding variable. In the following two subsections we look at the two quality measures naturalness and comprehensibility, and analyze whether there were differences between the two groups that could have influenced the differences in ratings of uncertainty.

##### 3.3.2.1 Naturalness

**Figure 5:**
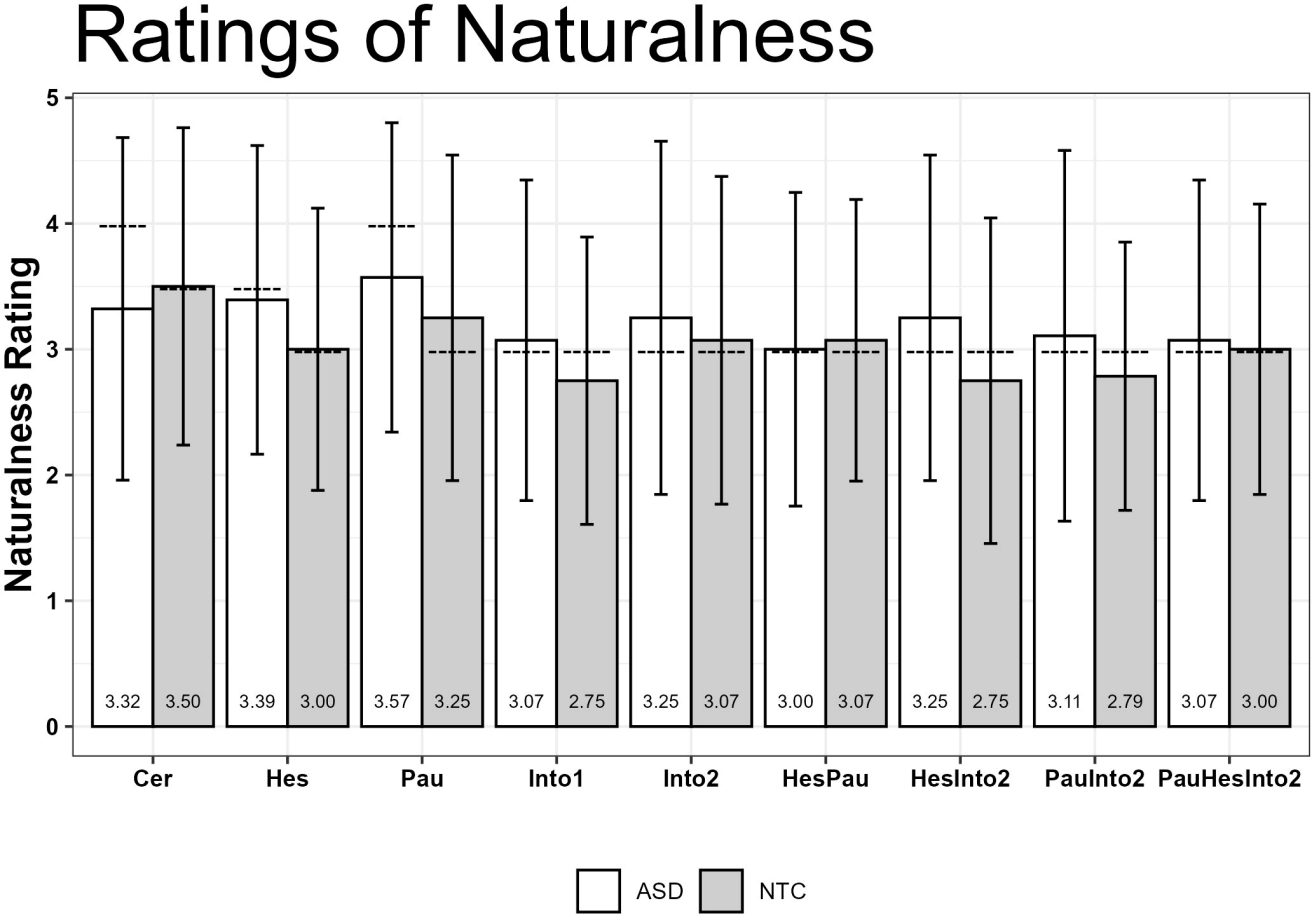
Means of naturalness ratings (1=little natural, 5=very natural), dashed lines denote the median.

The statistical analysis of the naturalness ratings using the robust ANOVA revealed neither significant main effects nor a significant interaction (main effect “diagnostic group”: robust test statistic *F*(1, 32.872) < 1; main effect “prosodic uncertainty condition”: *F*(7, 24.970) = 2.24, *p* = .065; interaction: *F*(7, 24.970) < 1). In **Figure 5**, means of naturalness ratings are depicted for all factorial combinations. Further exploratory analyses for the prosodic uncertainty conditions revealed that the largest difference between conditions was found for the contrast Cer-PauInto2, which had the highest/lowest mean naturalness ratings (Cer: *M* = 3.41, *SD* = 1.30, PauInto2: *M* = 2.95, *S*D = 1.26; robust test statistic = 3.19, *p* = .003, ES = 0.28).

The robust analysis of response times for naturalness ratings showed a significant main effect of “prosodic uncertainty condition”: *F*(7, 24.643) = 3.33, *p* = .012), whereas the main effect “diagnostic group” and the interaction were far from being significant (robust test statistic *F*(1, 32) < 1 and *F*(7, 25) < 1, respectively). Further exploratory analyses for the prosodic uncertainty conditions revealed that the largest response time difference between conditions was noted for the contrast Cer-PauHesInto2 that had the highest/lowest mean response times (Cer: *M* = 3155, *SD* = 2300, PauHesInto2: *M* = 4383, *SD* = 2922; robust test statistic = -3.70, *p* <.001, ES = 0.44).

##### 3.3.2.2 Comprehensibility

**Figure 6:**
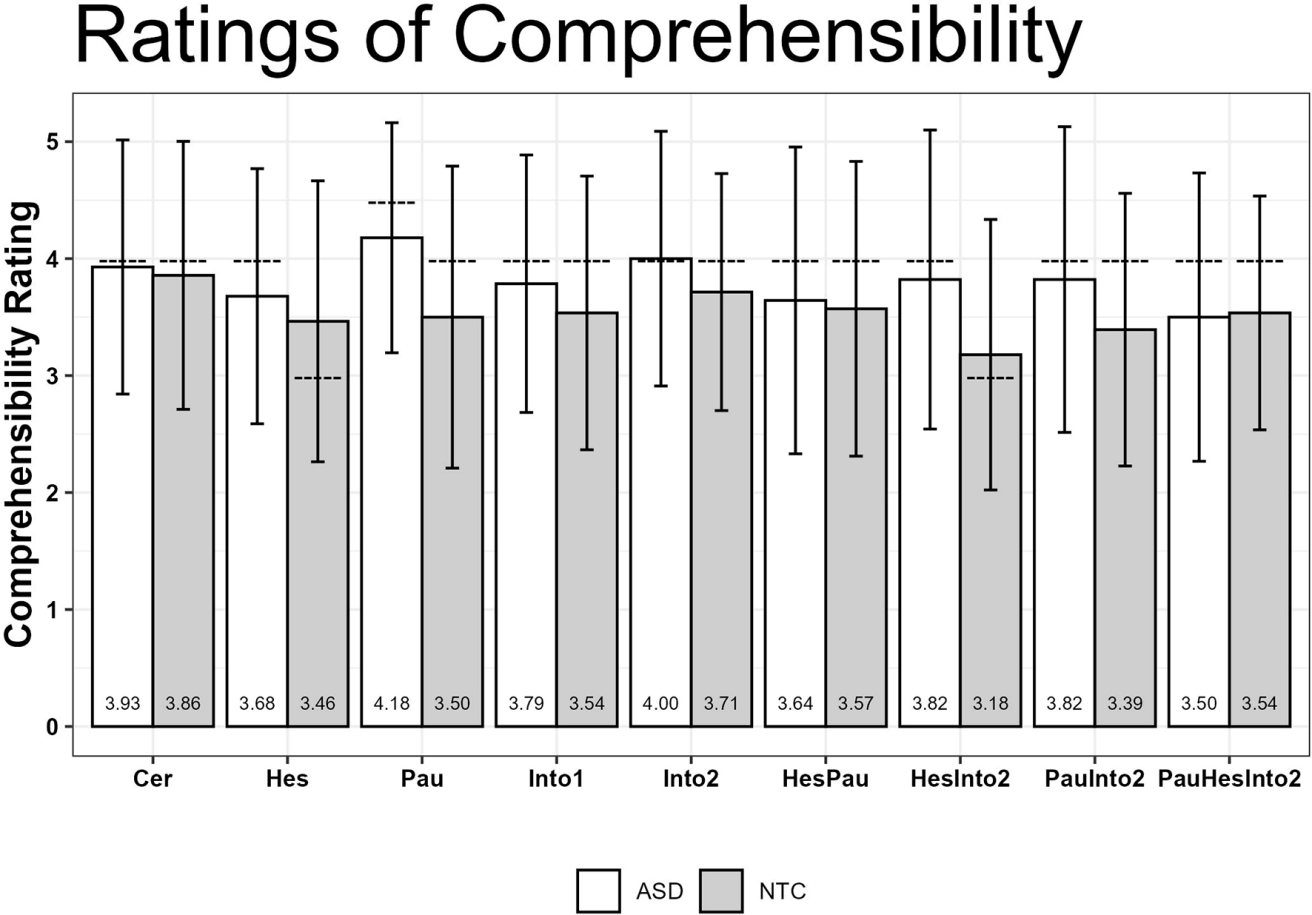
Means of comprehensibility ratings (1=little comprehensible, 5=very comprehensible), dashed lines denote the median.

Statistical analysis for the ratings of comprehensibility using the robust ANOVA revealed neither significant main effects nor a significant interaction (main effect “diagnostic group”: robust test statistic *F*(1, 32.647) = 3.21, *p* = .082; main effect “prosodic uncertainty condition”: *F*(7, 24.887) = 1.22, *p* = .331; interaction: *F*(7, 24.887) < 1). In **Figure 6**, means of comprehensibility ratings are shown for all factorial combinations. Further explorative analyses for the prosodic uncertainty conditions revealed that the biggest difference between conditions was noted for the contrast Cer-PauHesInto2, which had the highest/second lowest mean naturalness ratings (Cer: *M* = 3.89, *SD* = 1.11, PauInto2: *M* = 3.52, *SD* = 1.11; robust test statistic = 2.54, *p* = .016, ES = 0.25).

The robust analysis of response times for comprehensibility ratings showed a significant main effect of “prosodic uncertainty condition”: *F*(7, 24.834) = 6.59, *p* = .0002), whereas the main effect “diagnostic group” and the interaction were not significant (robust test statistic *F*(1, 29.434) = 1.10, *p* = .303 and *F*(7, 24.834) = 1.858, *p* = .120, respectively). Further exploratory analyses for the prosodic uncertainty conditions revealed that the largest response time difference between conditions was noted for the contrast Cer-PauHesInto2, which had the second highest/lowest mean response times (Cer: *M* = 3013, *SD* = 1975, PauHesInto2: *M* = 4608, *SD* = 3341; robust test statistic = -4.84, *p* <.0001, ES = 0.44).

We summarize the results for the assessment of the perceived naturalness and comprehensibility of the stimuli as follows: Our data show no significant group difference with respect to either dimension.

##### 3.3.2.3 Correlation between perceived uncertainty and naturalness or comprehensibility

Fisher’s z-transformed correlations were calculated to test the relationship between perceived uncertainty and naturalness as well as perceived uncertainty and comprehensibility [see (**Table 7**)]. Significant group differences were found between the correlation of uncertainty and naturalness, i.e. in the ASD group the correlation is significantly lower than in the NTC group. This means that the processing of naturalness and uncertainty are more closely linked in the NTC group than in the ASD group which may indicate a different type of processing in ASD.

**Table 7:** Fisher’s z transformed correlations for uncertainty and naturalness and also for uncertainty and comprehensibility.

	Fisher’s z transformed correlations							
	ASD			NTC			*t-test*	*p*
	*n*	*M*	*SD*	*n*	*M*	*SD*		
Uncertainty - Naturalness	27 ***^a^***	0.209	0.470	28	0.474	0.487	-2.055	.045
Uncertainty - Comprehensibility	27 ***^a^***	0.219	0.616	27 ***^b^***	0.360	0.461	-0.949	.348

Note. **^a^** One person in the ASD group gave the same score for all ten different levels of certainty. Due to the lack of variability, correlations cannot be calculated for this person. **^b^** One person from the NTC group gave the same scores for certainty and comprehensibility, resulting in a correlation of 1.0. Therefore, the correlation for this person could not be transformed to Fisher’s z and had to be excluded from the analysis.

## 4 Discussion

### 4.1 Summary

In this study, we experimentally investigated how prosodic cues of uncertainty were perceived by hearers with ASD without II in comparison to a NTC group. The synthetic utterances were generated by an articulatory speech synthesizer (33). They were embedded in short question-answer pairs using a scenario in which the question was asked by a human in a natural voice. The robot gave a synthetic response in which the cues pause, intonation and hesitation were varied to generate different levels of intended uncertainty. The synthetic responses were rated by participants on rating scales for (i) uncertainty, (ii) naturalness, and (iii) comprehensibility. Reaction time of the rating was also measured. In addition, a complementary audiometric test, a pitch discrimination test and a pitch change test were performed.

The results for the level *Hesitation* combined with *Intonation 2* showed a group difference in reaction times to judge uncertainty perception: the ASD group took longer for the judgment than the NTC group. Note that this difference is no longer significant after Bonferroni correction. All other levels of uncertainty were not reliably different between the two groups (all *p*s >.10). With the exception of pause, all judgments were reported as more certain in average in the ASD group. In addition, the intended levels of uncertainty showed a tendency for longer reaction times in the ASD group.

No significant difference was found between the ASD and NTC groups in the pitch discrimination and pitch change task for baseline discrimination. Although pitch differences were perceived equally well, the prosodic cues tended to be interpreted differently in terms of uncertainty perception: the intended prosodic cues of uncertainty influenced the perception of hearers less in the ASD group than in the NTC group. However, the ASD group showed longer reaction times than the NTC group. A possible explanation could be that a higher cognitive load was required for the hearers with ASD without II. It is assumed that hearers in the NTC group processed the prosodic cues automatically and with less cognitive effort, allowing them to make their ratings of uncertainty more quickly.

The differential correlation effect, i.e. that ASD individuals show a lower correlation between their ratings of naturalness on the one hand and uncertainty on the other, can be taken as evidence that the co-processing of naturalness and uncertainty is not as tightly linked in the ASD group compared to the typical co-processing of uncertainty cues with the naturalness of the utterance. This weaker relationship would be consistent with weak central coherence accounts of autism [e.g. (10)].

### 4.2 Limitations and future directions

Due to the design of the study, there were only few observations per participant, which means that in the current study there were significantly fewer trials per condition in the responses to uncertainty perception than in the pilot study (109). It is possible that the few observations from participants and the resulting study design had an impact on the results and could explain the non-significant differences in uncertainty judgments between the ASD group and the NTC group in our data. The reason for presenting only a subset of the stimuli to the participants was to minimize learning effects of participants. Previous experimental research on the role of prosody in pragmatic focus interpretation and possible learning effects is described in Fisseni (30), and Wollermann (51).

As a consequence of our feasibility study, the number of trials per condition could be increased and the test conditions could be adjusted in order to collect more data and verify the results. We could also reduce the number of psychological and psychiatric tests in order to save time and cognitive capacity. In particular, the tests for minimal pitch discrimination and pitch change could be omitted, as we have not found correlations between baseline auditory abilities and prosody perception in ASD. Instead, we would like to focus on the presentation of the critical stimuli for uncertainty perception by further minimizing learning effects.

It is possible that the order of presentation of the stimuli has an effect on recipients’ judgments, i.e. the stimulus presented first may be judged differently from stimuli presented later due to possible learning effects. Furthermore, in future research, we could focus on testing hearing abilities by including a group of participants with hearing impairments for comparison with the ASD and NTC groups.

In our approach, we used synthetic speech to generate the different utterances with intended uncertainty. In this way, specific prosodic parameters could be manipulated while the influence of unintended variation was minimized compared to natural speech, so we gave high priority to controllability and selective manipulation. A possible explanation for the non-significant effects of prosody on uncertainty perception could be the use of synthetic speech. It is conceivable that the effects of prosody on uncertainty perception might be more evident when natural speech is used. However, natural speech is less controllable than synthetic speech. In future work, it may be an option to consider neural synthesizers in comparison to our articulatory speech synthesizer for similar experiments. However, our primary goal was to achieve manipulability and controllability. It is an open question to what extent this can be guaranteed by neural synthesis.^12^

In future experiments we would like to further exploit the advantages of speech synthesis. In particular, we would like to explore the interplay between the three acoustic cues pause, intonation and hesitation to model different degrees of uncertainty in more detail. For example, it would be interesting to test a duration <4 seconds for the pause and also to use other hesitation particles besides uh, such as um. We also think it is important to experimentally investigate the role of lengthening in uncertainty perception, as pointed out by Betz et al. (93).

Another limitation is the material used in this study. We designed short question-answer situations in order to not only present the synthetic stimuli without embedded context. The answers were one word sentences. In future work, it would be important to test more complex sentences for ecological validity. However, it should be noted that the dialogues were simulated and did not approximate real-life dialogues, which induce uncertainty. This may have influenced the pattern of the results, i.e., the lack of significant interactions of the different variables with the group.

Next, we tested only adult participants. A wider range of ages, especially children and adolescents, might provide more information about the developmental trajectories of the ability to adequately process prosodic cues of uncertainty. Krahmer and Swerts (61) tested 7-8 year old neurotypical children and adults on the perception and production of uncertainty in question-answer situations. Uncertain utterances produced by adult speakers were recognized more accurately by both children and adult hearers than uncertain utterances produced by children. In addition, adults performed better than children in the recognition of uncertainty. We therefore plan to conduct further studies with children and adolescents. It should be noted that it would be necessary to modify the methodological approach with regard to cognitive abilities, especially in the case of children.

At this point, we would like to take a critical look at the role of ToM for prosody perception. For prosody processing, it may be important whether the ToM is built up automatically in an incidental or cognitive-compensatory manner, as we have explained above. If we assume that prosody perception supports the construction of ToM, there may also be incidental and compensatory prosody processing. This could explain differences in reaction times.

In addition, we only looked at individuals with ASD without II, so it is not possible to generalize the results to all individuals with a diagnosis of ASD.

In future work, we would like to conduct further perceptual experiments of affective prosody recognition, as the investigation of speech characteristics may display a promising novel biomarker and may contribute to the better understanding of mental disorders [cf. (117): 337; see also (118): 99].

## Data Availability

The original contributions presented in the study are included in the article/**Supplementary Material**. Further inquiries can be directed to the corresponding author.

## Footnotes

1 Our own empirical studies refer only to German. However, we assume that other West Germanic languages work in a very similar way. Studies on other languages are cited for methodological reasons.

2 Fisseni (30) provides a systematic overview of the term *focus*; for a model of pragmatic focus interpretation see also Wollermann et al. (31).

3 It should be noted that we are referring to the current version of the VocalTractlab website (33).

4 A description of the false-belief task can be found in Wimmer and Perner (68). A brief overview of the different levels of ToM, i.e. first-order ToM (e.g., “X thinks or feels…”), second-order ToM (e.g., “X thinks that Y feels…”), and third-order ToM (e.g., “X believes that Y assumes that Z intends …”) can be found in Gabriel et al. [ (69): 534-35]. The ToM of a subject S is generally understood as S's beliefs about mental states, such as beliefs, intentions, or emotions, of another subject O. If S's beliefs concern O's beliefs about mental states of subjects O_2 other than O, we speak of second-order ToM. O_2's mental states can also be beliefs about mental states of other persons. In this case we speak of third-order beliefs.

5 According to Begeer et al. (75) counterfactual reasoning describes a phenomenon in which people imagine alternatives to one or more features of a perceived event [see also (76)]. It can be characterized by switching back and forth between a real situation and an imagined situation, i.e. a so-called counterfactual situation.

6 For a more detailed discussion see Gyarmathy and Horváth [ (90): 27].

7 For a detailed discussion of the role of uncertainty in human-machine-interaction, see Wollermann [ (51): 91].

8 Unit selection is a method for acoustic speech synthesis based on large corpora of naturally spoken utterances. Units are selected with respect to a target utterance by means of concatenation. The generated speech is characterized by high comprehensibility [cf. (101): 279].

9 It should be noted that in our current study we also measure comprehensibility in order to better understand the processing of synthetic utterances.

10 It should be noted that the articulatory speech synthesizer is regularly updated. In the following we refer to the Vocaltract Lab website (33), but in our previous studies we have used older versions of the system.

11 A strong pause of 4 s was used because we could not find exact values for pauses in the literature for modeling uncertainty in question-answer situations in human-machine communication [cf. (109): 40]. It was important for us to use very clear characteristics of uncertainty to test whether there are effects. We have already successfully used this value of 4 s in our pilot study (109).

12 An overview of neural text-to-speech synthesis is given by Tan (116).
