# Supplementary material for: Processing of prosodic cues of uncertainty in autistic and non-autistic adults: a study based on articulatory speech synthesis
Source: Front Psychiatry. 2024 Oct 14;15:1347913. doi: 10.3389/fpsyt.2024.1347913 (PMC11513626; doi:10.3389/fpsyt.2024.1347913)
Supplement: Supplementary file 1 [file Table1.docx]

**Appendix: Experimental Design**

*Abbreviations for intended uncertainty levels are explained in Table 1. Distr: Distractor*

| **Pos.** | **Group 1 (A)** | **Group 2 (B)** | **Group 3 (C)** | **Group 4 (D)** |
| --- | --- | --- | --- | --- |
| 1 | Distr;Lime | Distr;Lime | Distr;Lime | Distr;Lime |
| 2 | Into2;Melons | Distr;Cucumbers | Distr;Cucumbers | Cer;Tomatoes |
| 3 | Distr;Beans | Hes;Raisins | Into1;Bananas | PauHesInto2;Tomatoes |
| 4 | Distr;Strawberries | Cer;Bananas | HesPau;Tomatoes | Distr;Paprika |
| 5 | PauHesInto2;Melons | Distr;Pears | Distr;Strawberries | HesPau;Melons |
| 6 | Into1;Tomatoes | PauHesInto2;Bananas | Hes;Tomatoes | Distr;Beans |
| 7 | Distr;Paprika | HesInto2;Tomatoes | Distr;Garlic | Distr;Blueberries |
| 8 | HesInto2;Raisins | Distr;Mandarins | Distr;Mandarins | PauInto2;Raisins |
| 9 | Distr;Blueberries | Distr;Beans | Pau;Melons | Distr;Garlic |
| 10 | PauInto2;Tomatoes | Into1;Melons | HesInto2;Melons | Into2;Tomatoes |
| 11 | Distr;Mandarins | Distr;Paprika | Distr;Paprika | Pau;Bananas |
| 12 | Cer;Melons | Into2;Bananas | PauInto2;Bananas | Distr;Mandarins |
| 13 | Distr;Cucumbers | Distr;Blueberries | Distr;Blueberries | Into1;Raisins |
| 14 | Distr;Pears | Pau;Tomatoes | Distr;Pears | Distr;Pears |
| 15 | Pau;Raisins | Distr;Oranges | PauHesInto2;Raisins | Distr;Cucumbers |
| 16 | Distr;Garlic | PauInto2;Melons | Cer;Raisins | HesInto2;Bananas |
| 17 | Distr;Oranges | HesPau;Raisins | Distr;Oranges | Distr;Strawberries |
| 18 | Hes;Bananas | Distr;Garlic | Into2;Raisins | Hes;Melons |
| 19 | HesPau;Bananas | Distr;Strawberries | Distr;Beans | Distr;Oranges |
